# Employment at the onset of partial disability retirement and four years later

**DOI:** 10.1093/eurpub/ckac130.129

**Published:** 2022-10-25

**Authors:** A Polvinen

**Affiliations:** The Finnish Centre for Pensions, Helsinki, Finland

## Abstract

**Background:**

Increasing the labour market participation of people with a reduced ability to work is a big labour market challenge in many countries. In Finland, one third of all new disability pensions are granted as partial pensions. It is also known that most partial disability pensioners continue working while receiving a pension. There is only limited research about the length of employment of partial disability pensioners and the factors associated with employment in retirement. The aim of this study was to examine the employment at the onset of partial disability retirement and four years after retirement, and to examine how age, gender, educational level, employment sector, diagnosis and type of pension are associated with employment after partial disability retirement.

**Methods:**

Our Finnish register data comprised 7,617 partial disability pensioners aged 20-58 who retired in 2013 or 2014. Logistic and multinomial logistic regression analysis were used to estimate OR:s and 95 percent CI:s for employment at the onset of partial disability retirement and four years after retirement.

**Results:**

81 percent of partial disability pensioners were employed when retiring on a partial disability pension. Four years later, 50 percent were still drawing a partial disability pension, of whom 77 percent were working. 11 percent were employed but no longer drawing a partial disability pension. The partial disability pensioners who were more often employed were younger, female, more highly educated, working in the public sector and receiving a temporary disability pension.

**Conclusions:**

Many sociodemographic factors are associated with employment at the onset of partial disability retirement and with continued working while drawing a partial disability pension.

**Key messages:**

- Employment after partial disability retirement is common and relatively stable. After four years of retirement, more than 70 percent of partial disability pensioners were still working.
- Many sociodemographic factors are associated with employment after partial disability retirement.

